# Glycolipid Biosurfactants in Skincare Applications: Challenges and Recommendations for Future Exploitation

**DOI:** 10.3390/molecules28114463

**Published:** 2023-05-31

**Authors:** Simms A. Adu, Matthew S. Twigg, Patrick J. Naughton, Roger Marchant, Ibrahim M. Banat

**Affiliations:** 1 The Nutrition Innovation Centre for Food and Health (NICHE), School of Biomedical Sciences, Faculty of Life and Health Sciences, Ulster University, Coleraine BT52 1SA, UK; adu-s@ulster.ac.uk (S.A.A.); pj.naughton@ulster.ac.uk (P.J.N.); 2 Pharmaceutical Science Research Group, Biomedical Science Research Institute, Ulster University, Coleraine BT52 1SA, UK; m.twigg@ulster.ac.uk (M.S.T.); roger.marchant@ulster.ac.uk (R.M.)

**Keywords:** biosurfactants, glycolipids, sophorolipids, rhamnolipids, synthetic surfactants, purification, biosurfactant characterisation, skincare, bioassays, bioactivities, skin cells, 3D in vitro skin model

## Abstract

The 21st century has seen a substantial increase in the industrial applications of glycolipid biosurfactant technology. The market value of the glycolipid class of molecules, sophorolipids, was estimated to be USD 409.84 million in 2021, with that of rhamnolipid molecules projected to reach USD 2.7 billion by 2026. In the skincare industry, sophorolipid and rhamnolipid biosurfactants have demonstrated the potential to offer a natural, sustainable, and skin-compatible alternative to synthetically derived surfactant compounds. However, there are still many barriers to the wide-scale market adoption of glycolipid technology. These barriers include low product yield (particularly for rhamnolipids) and potential pathogenicity of some native glycolipid-producing microorganisms. Additionally, the use of impure preparations and/or poorly characterised congeners as well as low-throughput methodologies in the safety and bioactivity assessment of sophorolipids and rhamnolipids challenges their increased utilisation in both academic research and skincare applications. This review considers the current trend towards the utilisation of sophorolipid and rhamnolipid biosurfactants as substitutes to synthetically derived surfactant molecules in skincare applications, the challenges associated with their application, and relevant solutions proposed by the biotechnology industry. In addition, we recommend experimental techniques/methodologies, which, if employed, could contribute significantly to increasing the acceptance of glycolipid biosurfactants for use in skincare applications while maintaining consistency in biosurfactant research outputs.

## 1. Introduction

Microbial biosurfactants are surface-active compounds of biological origin, sustainably produced as secondary metabolites by bacteria, yeast, and filamentous fungi [1,2,3]. Biosurfactants are amphiphilic in nature; therefore, they are able to interact at both hydrophilic and hydrophobic interfaces [4,5]. The hydrophilic moiety of a biosurfactant molecule may be composed of carbohydrates, amino acids, phosphate, cyclic peptides, or carboxylic acids. Conversely, the hydrophobic component of these molecules is mostly made of hydroxyl fatty acids, long-chain fatty acids, or α-alkyl-β-hydroxy fatty acids [6,7,8]. The diversity in structure and chemical composition of biosurfactants results from their microbial origin, culture medium, substrates utilised during microbial fermentation, and physical cultivation conditions [7,9,10]. Consequently, biosurfactants are categorised based on their microbial origin and chemical composition [11,12]. The major classes of biosurfactants include glycolipids, lipopeptides, phospholipids, lipoproteins, and polysaccharide–protein–fatty acid complexes [5,13,14]. Glycolipid biosurfactants constitute the most commercially exploited group of biosurfactants, and these molecules will be the focus of this review [13,15,16,17].

Glycolipid biosurfactants consist of carbohydrate moieties linked to long-chain aliphatic acids or hydroxy aliphatic acids of varying lengths [16,18,19]. The difference in carbohydrate moiety and fatty acid chains accounts for the diversity and subclasses of glycolipids, which include sophorolipids, rhamnolipids, trehalolipids, and mannosylerythritol lipids [16]. The most intensively studied groups of glycolipids are sophorolipids mainly produced by the yeast species *Starmerella bombicola* often as a crude mixture of acidic and lactonic forms and rhamnolipids mainly produced by the Gram-negative bacterium *Pseudomonas aeruginosa* as a crude mixture of mono-rhamnolipid and di-rhamnolipid congeners [2,20]. The biosynthetic processes, structural composition, and fermentation process/conditions of sophorolipids and rhamnolipids have been extensively investigated and reported [11,21,22,23,24,25].

Generally, sophorolipids and rhamnolipids are good at reducing surface and interfacial tension of solid–liquid, liquid–liquid, and liquid–gas interphases and, as a result, produce excellent emulsification, wetting, solubilisation, and detergency functions [13,26,27,28,29]. In addition, sophorolipids and rhamnolipids have been reported to have a number of compelling advantages over their chemical counterparts; these advantages include high rate of biodegradability; potential compatibility with human skin often investigated via in vitro cytotoxicity studies; and production from potentially cheap and renewable substrates sourced from waste material such as animal fat, waste oil, glycerol, and whey waste [19,30,31]. Furthermore, the potential added functionalities of these glycolipid biosurfactants such as antimicrobial effects on skin pathogens, anticancer effects against human malignant melanocytes, antiaging/antiwrinkle effects on dermal fibroblasts, wound healing, and immunomodulatory effects on a monolayer of skin cells in vitro have been reported in several studies [27,32,33,34].

The popularity of sophorolipid and rhamnolipid biosurfactants and their potential advantages over petrochemically derived synthetic surfactants are reflected in the comparable increase the in number of publications in recent years on their production, characterisation, and assessment of bioactivities. Consequently, sophorolipids and rhamnolipids are gaining significant traction for use in the food, pharmaceutical, and skincare industries [12,19,35,36,37,38]. However, low product yield and sophisticated production processes alongside limited structural variability are among the paramount challenges affecting the utilisation of these biosurfactants in academic and industrial research, especially for the skincare and pharmaceutical applications [3,21,27,39,40,41,42]. Additionally, in the bioactivity assessment of sophorolipids and rhamnolipids, most studies lack sufficient comparative experimental controls such as the use of a synthetic surfactant, whose effects on skin cells or skin bacteria can be compared directly with these glycolipids in studies where the investigations of suitable natural and skin-compatible alternatives to synthetic surfactants for potential skincare applications is a priority [27,42]. Furthermore, most in vitro studies on the effects of these glycolipids on skin cells and bacteria were conducted using 2D in vitro cell cultures, thus, the inability of these cell culture models to accurately represent the in vivo human skin and the skin microenvironment [31,42]. Nevertheless, in recent years, the use of metabolic engineering, microbial enzymes, optimisation of cell culture media and fermentation techniques, utilisation of purified and chemically characterised glycolipid congeners in bioassays, and 3D in vitro cultures have been the emerging strategies to overcoming the above-mentioned obstacles in biosurfactant research and applications [12,21,24,35,43]. These initiatives would allow for an appropriate substantiation and validation of the efficacy and differential bioactivities of individual glycolipid congeners, effectively mimicking the complexity of in vivo systems in bioassays, and ultimately making glycolipid biosurfactants attractive for use in skincare applications.

This review, therefore, aims to provide comprehensive information on the current trend towards the utilisation of sophorolipid and rhamnolipid biosurfactants as potential substitutes to synthetic surfactants in skincare applications, challenges associated with the use of these glycolipid biosurfactant molecules in bioassays, and recommendations for their future exploitation for use in the skincare industry.

## 2. Glycolipid Biosurfactants as Promising Alternatives to Synthetic Surfactants

### 2.1. Adverse Effects of Synthetic Surfactants on the Environment and Consumer Skin Health

Surfactants are a principal ingredient of cosmetic and personal care products with specialised abilities to reduce surface and interfacial tension of fluids, solubilise and emulsify additional skincare ingredients, and improve/stabilise foam/gel formation [44,45]. The current global market size of surfactants stands at approximately USD 42.1 billion, and this is projected to increase by 80% by the end of 2025 [46]. However, it is likely that this predicted market size will exceed the conservative estimate considering the increased consumer demand and consumption of skincare products, such as hand sanitisers, which have seen a substantial increase in use resulting from the SARS-CoV-2 viral pandemic [46]. At present, most of the surfactants utilised in skincare products are chemically synthesised from non-sustainable and poorly degradable petrochemical resources [3,38,45,47,48]. Examples of commercially available synthetic surfactants utilised in current skincare products include cocamidopropyl betaine, cocamide diethanolamide, sodium dodecyl sulphate (SDS), and sodium lauryl ether sulphate (SLES) [49,50].

Although these synthetic surfactants have been reported to be effective in several skincare products, there are growing concerns that their low rate of biodegradability and increased toxicity pose significant risks to the health of consumers and the environment [6,46,47,51]. For instance, after domestic applications (e.g., body wash, laundry, etc.), the entry of these synthetic surfactants into bodies of water via domestic wastewater treatment plant effluents is an emerging concern with regard to fish kills and water pollution [46,52,53]. Although there are techniques for treatment (e.g., bioadsorption and biodegradation of surfactants on activated carbon source), the high concentrations of synthetic surfactants (1–10 mg L^−1^) often found in domestic wastewater during treatments, coupled with their low rate of biodegradability, fail almost all conventional treatment methods, hence posing significant health risks to the aquatic ecosystem [46,54]. Moreover, the prolonged use of skincare products formulated with synthetic surfactants has been reported to potentially disrupt the human skin microflora, cause skin irritations and allergic reactions, and disrupt the skin barrier integrity [44,49,55,56,57]. An epidemiological survey in the UK revealed that in a year, about 23% of females and 14% of males typically experience some form of adverse effects after the application of cosmetic and personal skincare products, 10% of which are allergic reactions [45,58]. It is, therefore, a priority to investigate more biodegradable, biocompatible, and sustainable alternatives to synthetic surfactants that can be utilised in skincare applications without compromising on the quality and/or the efficacy of skincare products. Such a promising alternative has been the use of glycolipid biosurfactants, considering their several potential advantages over synthetic surfactants [6,11,37,38,45].

### 2.2. Effects of Glycolipid Biosurfactants on Various Human Skin Cell Types

There have been several studies demonstrating the effects of glycolipid biosurfactants on various skin cell types as a means of assessing their potential use in skincare applications [19,27,59]. In 2018, Maeng et al. [27] evaluated the cytotoxicity, antiwrinkle, wound healing, and immunomodulatory effects of mixed sophorolipid congeners produced via the fermentation of hydrolysed horse oil on human dermal fibroblastic cells. The authors demonstrated that concentrations of mixed sophorolipid congeners ranging from 1 to 10 μg mL^−1^ stimulated the expression of the type 1 collagen gene *Col-1* while inhibiting elastase enzymes, both of which are key contributors to the prevention of skin aging [27]. Moreover, treatment concentrations up to 50 μg mL^−1^ of sophorolipids had no significant effects on the viability of the dermal fibroblastic cells [27]. In addition, 5–25 μg mL^−1^ sophorolipid treatments significantly attenuated the mRNA expression levels of *IL-6*, *TNF-a*, and *COX-2* in the lipopolysaccharide-treated murine macrophage cell line RAW 2647 [27]. This potentiates the immunomodulatory effects of sophorolipids and, consequently, their incorporation into topical creams for the treatment of skin infections, such as psoriasis, which are often characterised by a massive accumulation of granulocytes and the hypergenesis of skin cells [27,60,61]. Furthermore, following the creation of artificial wound in the human fibroblastic cell lines and subsequent treatment with sophorolipids at concentrations ranging from 0.5 to 5 μg mL^−1^, Maeng et al. (2018) [27] demonstrated that the sophorolipid treatment significantly improved the closure of the artificial wounds, thus demonstrating potential in vitro wound healing effects. Wound healing is a complex biological process involving different epidermal and dermal cells, which are well coordinated by a myriad of immune cells and growth factors [62,63]. Despite the significant improvement in wound treatments in recent decades, skin grafting remains one of the commonest surgical procedures for treating chronic wounds [63,64,65]. Therefore, the in vitro wound healing effects of sophorolipid biosurfactants demonstrated by Maeng et al. (2018) [27] is a significant step towards the identification of potential novel natural ingredients for use in wound care formulations to accelerate chronic wound healing and to reduce reliance on skin grafting. In another study on the characterisation of the cytotoxic effects of sophorolipids by Lydon and colleagues [59], concentrations up to 0.5 mg mL^−1^ of acidic sophorolipids (Acidic SL) had no significant cytotoxic effects on either human umbilical vein endothelial cells, human dermal microvascular endothelial cells, or spontaneously transformed human keratinocytes (HaCaT cells) after 24 h. More recently, Manikkasundaram and colleagues [42] extracted a glycolipid biosurfactant from *Streptomyces enissocaesilis*, HRB1, characterising it to identify the molecular structure. Although the exact congener of glycolipid was not reported, the authors evaluated the biomedical and bioremediation potential of the glycolipid extract [42]. It was demonstrated that the crude glycolipid biosurfactant inhibited the formation of 71% *P. aeruginosa* biofilms at treatment concentrations of 512 μg mL^−1^ and exhibited antioxidant properties at 1000 μg mL^−1^ [42]. Additionally, the glycolipid biosurfactant reduced the viability of leukaemia (Jurkat) by 71.3% and myeloma (H929) cancer cell lines by 68.5% at a concentration of 100 μg mL^−1^ while reducing the viability of human normal dermal fibroblastic cells (HDF) to 56.8% only at treatment concentrations exceeding 250 μg mL^−1^ [42]. Clearly, these biological activities of sophorolipids warrant their potential use in antiaging, anticancer, wound healing, sunscreen, and skin moisturising formulations. At present, companies such as Givaudan in France, Holiferm in the UK, and Kanebo skincare in Japan commercially produce sophorolipids for use in the formulation of cosmetic and personal care products, such as deodorants, shower gels, lipsticks, and skin and hair moisturisers [45,66,67].

As with sophorolipids, assessments of the potential use of rhamnolipids in cosmetic and personal care formulations have been carried out in a number of studies [20,23]. The cytotoxicity profile of rhamnolipids extracted from *Marinobacter* MCTG107b and *Pseudomonas* MCTG214 (3b1) strains was evaluated using HaCaT and adult liver epithelial (THLE 3) cells via propidium iodide and alamar blue assays [31,68,69]. The biosurfactants exhibited negligible cytotoxic effects on both cell lines after 72 h of treatment with up 0.25 mg mL^−1^ concentrations of rhamnolipid [31]. Conversely, the synthetic surfactants SLES and sodium lauroylsarcosinate significantly reduced cell viability at concentrations less than 0.002 mg mL^−1^ [31]. Based on these findings, the authors suggested that rhamnolipids could potentially offer a biocompatible alternative to commercially available synthetic surfactants [31]. Haque and colleagues [64] also investigated the antioxidant capacity of rhamnolipids produced by the *P. aeruginosa* MN1 strain using 1-diphenyl-2-picrylhydrazyl radical scavenging assays (DPPH). Using ascorbic acid as positive control, the rhamnolipids exhibited substantial radical scavenging activities in a concentration-dependent manner with maximum activity observed at concentrations of 5 mg mL^−1^ [70]. Rhamnolipids and sophorolipids sourced from Evonik Industries were utilised as emulsifiers in the development of sustainable lip gloss formulations [71]. Their results showed that both rhamnolipids and sophorolipids demonstrated excellent formulation-stabilising properties when aqueous solutions of rhamnolipids and sophorolipids were mixed into silicone oil, resulting in the formation of stable and transparent systems [71]. Although there were no effects of the glycolipids on the rheology of the lip gloss formulation, rhamnolipids and sophorolipids could be used as emulsifiers in lip gloss while investigating other compounds such as large silica particles to improve the viscosity of lip gloss formulations [71].

### 2.3. Effects of Glycolipid Biosurfactants on the Human Skin Microbiome

The human skin microbiome is a collection of diverse bacteria, viruses, and fungi indigenously colonising the human skin [72,73,74]. These resident skin microbes are found within different niches of the skin epidermis, including the dry (legs and arms), moist (armpit, antecubital fossa, and toe web space), and sebaceous areas (face, upper chest, retroauricular crease, back, occiput, and glabella) [72,75,76]. Although some opportunistic pathogenic microorganisms may be present occasionally, recent studies in the fields of dermatology and microbiology have revealed that the skin microbiome of a healthy individual is composed of both commensal and mutualistic microorganisms with bacteria cells being the most predominant and most studied microorganisms [77]. Common representatives of the bacterial skin microbiota include *Staphylococcus*, *Micrococcus*, *Streptococcus*, and *Brevibacterium* species [73]. The interactions between the human skin microbiota, the skin tissue, and associated immune cells help maintain a healthy skin microbiome, preventing colonisation by pathogens [76]. Nevertheless, several factors lead to skin microbiome imbalance (dysbiosis) and, consequently, the onset of skin infections, such as atopic dermatitis, psoriasis, and eczema [78,79,80]. Dysbiosis in the skin microbiome may be caused by either intrinsic (inevitable, i.e., biologically, physiologically, or genetically determined) or extrinsic factors, such as diet, UV exposure, drugs, and the optional application of cosmetic and personal care products [81,82,83]. Thus, an important factor to be considered when formulating cosmetic and personal care products is the effects of the individual ingredients on the human skin and the skin microbiome [84].

Glycolipid biosurfactants have been hypothesised to potentially encourage the maintenance of the skin’s acidic pH as triglycerides in the fatty acid chain(s) of glycolipids congeners are hydrolysed by lipophilic skin commensals, such as *Cutibacterium acnes*, into free fatty acids [85,86,87,88]. Additionally, the fatty acids in glycolipids have been postulated to moisturise rough/dry skin surfaces and to act as antioxidants, thereby inhibiting the generation of free radicals when the human skin is exposed to UV radiation [45,85].

The human skin surface is sufficiently endowed with natural inhibitory substances, such as enzymes, bacteriocins, micrococcin, and alpha/beta defensins [74,89]. These natural inhibitory substances help to keep the skin microbiome in constant check against pathogens [51,89]. In this regard, several studies have demonstrated the antimicrobial effects of glycolipid biosurfactants against pathogenic skin bacteria, the majority of which are Gram-positive isolates [90,91]. It is worthy of note that the antimicrobial efficacy of glycolipid biosurfactants is dependent on their chemical structure and composition, treatment concentrations and duration of exposure to bacteria, and the class of bacteria under study [32,34]. Compared with non-acetylated acidic sophorolipids, monoacetylated and di-acetylated lactonic sophorolipids are more effective against Gram-positive pathogenic skin bacteria, such as *Staphylococcus aureus*, *Streptococcus pyogenes*, and *C. acnes*, which are the leading cause of atopic dermatitis (prevalent in 20% of children and 3% of adults worldwide), impetigo, and acne vulgaris, respectively [32,34,92]. Similar to sophorolipids, rhamnolipids have been demonstrated to be more effective against Gram-positive bacterial cells than Gram-negative isolates [91]. For instance, rhamnolipids treatment concentrations as low as 39.1 μg mL^−1^ are able to inhibit the growth of *S. aureus* after 24 h of exposure [93].

It is noteworthy that when glycolipid biosurfactants are utilised synergistically with conventional antibiotics, their efficacy is significantly improved, and as a result, they are able to inhibit Gram-negative nosocomial infective agents, such as *P. aeruginosa* and *Escherichia coli* [59,94,95]. Even so, current conventional antibiotics do not only inhibit the growth of pathogenic skin microbes, but rather at certain concentrations affect the healthy skin microbiome. This causes a delay in healthy skin microbiota restoration [96]. Consequently, there have been proposals to investigate the synergistic use and/or production of microbial glycolipid biosurfactants from non-pathogenic prebiotic (nutritional substances beneficial to the gut and skin microbiome) and probiotic (live microorganisms with health benefits) microorganisms, which will have the potential to encourage a healthy skin microbiome [96,97,98]. Lactic acid bacteria, such as those of the *Lactobacillus* spp., are well-known human intestinal-mucosa commensal bacteria utilised in most probiotic formulations [97,99]. Sharma and Singh [91] reported the simultaneous production of a glycolipid-type biosurfactant and bacteriocins from the cell culture supernatant of the *Lactobacillus casei* MRTL3 strain and tested their antimicrobial efficacy against eight pathogenic strains. Using agar diffusion assays, the bioactive compounds extracted from the *L. casei* MRTL3 strain were shown to have antimicrobial effects against both Gram-positive (*S. aureus*, *Bacillus cercus*, and *S. epidermidis*) and Gram-negative bacterial isolates (*P. aeruginosa*, *Salmonella typhi*) [97]. Similarly, using broth microdilution assay, Satpute et al. [94] reported the antimicrobial effects of glycolipids extracted from *L. acidophilus* NCIM 2903 stains against *E. coli*, *S. aureus*, *P. aeruginosa*, *B. subtilis*, and *Pseudomonas putida* at concentrations above 625 μg mL^−1^; bacterial viability was reduced to no less than 30%. For antibiofilm/antiadhesive assays, precoating medical-grade catheter and polydimethylsiloxane-based microfluidic channels with 625 μg mL^−1^ of the biosurfactant inhibited *P. vulgaris* and *B. subtilis* biofilm formation, respectively [100]. Studies such as those reported by Sharma and Singh [91] and Satpute and colleagues [94] are aimed at producing glycolipid biosurfactants from probiotic bacteria with antimicrobial effects and the potential to overcome the challenges of skin microbiome dysbiosis when the human skin is exposed to antimicrobial agents, thus making glycolipid biosurfactants more desirable for use in skincare and topical antimicrobial formulations [11,37,45,96,97,100,101]. A summary of the effects glycolipids elicit on the skin is provided in Table 1.

## 3. Challenges and Recommendations for Assessing the Potential Use of Glycolipid Biosurfactants in Skincare Applications

### 3.1. Pathogenicity of Glycolipid-Producing Strains, Low Product Yield, Cost of Large-Scale Production, and Limited Structural Variability

Skin surface moisturisation, cleansing, and protection of the human skin and the skin microflora are the distinctive features of effective skincare routines [102,103]. It is worth acknowledging that microbial glycolipid biosurfactants could only be utilised as a substitute to synthetically derived surfactants in skincare applications if they are able to deliver equal or better performance at a competitive market price [17,104]. *P. aeruginosa* is one of the most proficient producers of rhamnolipids [38,104,105]. However, the pathogenicity status of *P. aeruginosa* (biosafety level 2 pathogen) often associated with the production of toxins, such as pyocyanin, coupled with their low yield of biosurfactants makes them less attractive for industrial applications, particularly in food and skincare products [22,106]. Consequently, several methodologies to reduce/eliminate the toxicity of *P. aeruginosa* via the inhibition of pyocyanin biosynthesis and the investigation of novel microorganisms and bioprocessing techniques for rhamnolipid production are currently being exploited to improve the yield of rhamnolipids and their production from non-pathogenic strains [23,104,107]. To this end, Evonik Industries has investigated and reported the large-scale production of rhamnolipids from the genetically modified and non-pathogenic *P. putida* KT2440 strain [41,47,108]. Other bacterial isolates, including *Burkholderia thailandensis*, and genetically engineered yeast strains, such as *Saccharomyces cerevisiae*, are being investigated for yield optimisation following the successful production of rhamnolipids from these non-pathogenic strains [47,109]. In addition, the optimisation of fermentation conditions, such as temperature, dissolved oxygen, rate of aeration, and pH, have been demonstrated to be essential for improving the yield of rhamnolipids [41,110]. These yield optimisation measures together with the production of rhamnolipids from non-pathogenic microorganisms are a significant step to broadening the potential applications of rhamnolipids and increasing their acceptance for use in industrial applications, especially in the skincare industry, where biosurfactants are currently gaining significant attention for use as alternatives to synthetic surfactants [23,45,111].

Contrary to the pathogenicity status of the native-producing strain of rhamnolipids and their low yields, sophorolipid biosurfactants are majorly produced from non-pathogenic yeast strains at relatively high yields (up to 300 g L^−1^) and at a reduced production cost [24,25]. Hence, sophorolipids are considered the most commercially exploited class of glycolipid molecules [8,105]. The relatively high yield of sophorolipids as well as their low production cost is often attributed to the ability of native-producing strains to progress the biosynthesis of sophorolipids even at the cell-resting stage and the use of cheaper and readily available fermentation feedstocks. However, excessive foaming during the sophorolipid fermentation process resulting from culture aeration and agitation can cause a significant loss of products. Additionally, fermentation culture heterogenicity caused by the formation of a highly viscous second layer in cultures often reduces cellular exposure to oxygen and nutrients [40,112]. Nonetheless, the recent decade has seen the development of integrated sophorolipid production and separation techniques composed of foam fractioning, membrane, and gravity separation, which are capable of improving the recovery of sophorolipids after fermentation, even at an industrial scale [24,40].

**Figure 1:**
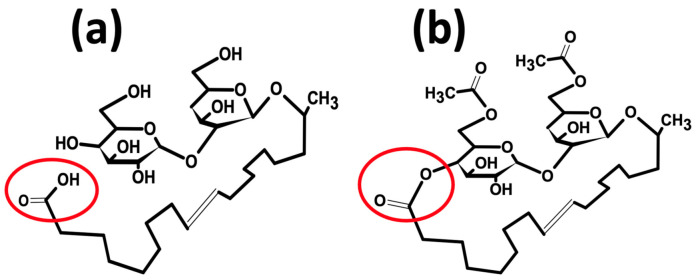
Chemical structure of (**a**) non-acetylated acidic sophorolipids (free fatty acid tail) and (**b**) di-acetylated lactonic sophorolipids (hydroxy fatty acid tail esterified at the 6′ or 6″ ends). Figure adapted from [19] and created with ChemSketch.com (accessed on 15 May 2023).

In addition to improving the product yield of glycolipids to make them economically viable for industrial applications, another challenge worth considering is the limited structural variability and, consequently, the low diversity of glycolipid-type biosurfactants in comparison with synthetic surfactants. The bioactivities and physiological properties of glycolipids are reported to be associated with their molecular structure; therefore, broadening up the structural variability of glycolipid biosurfactants could widen their bioactivities and physiochemical properties to allow for increased potential industrial applications [33,113]. The typical structure of sophorolipids is composed of a hydrophilic head (sophorose) bonded β-glycosidically to hydroxy fatty acids’ tail lengths of 16 or 18 [21]. The fatty acid moiety of sophorolipids may be free (acidic sophorolipids) or esterified at the C_4_″ end of the sophorose group (lactonic sophorolipids), whereas the hydrophilic (sophorose) group may be di-, mono-, or non-acetylated at the 6′ or 6″ end, depending on the producing strain (Figure 1) [25,114].

Approaches to broadening the structural diversity of sophorolipids include optimisation of fermentation and biosynthetic processes and enzyme-mediated chemical transformation [115,116,117,118]. For example, supplementation of fermentation culture with alkyl esters as hydrophobic substrate is reported to generate high proportion of acidic sophorolipids, whereas the use selective carbon sources, such as heptadecane and hexadecane, produces no less than 85% di-acetylated lactonic sophorolipids by native sophorolipid-producing strains [21,113]. Sophorolipids with a shorter fatty acid tail (12–16 carbon chain length) are highly desirability for use in cleansing products, considering their enhanced solubility and surface tension reduction capacity resulting from a balance in hydrophilic and hydrophobic moieties [119]. The use of already-hydroxylated substrates, such as hydroxyl fatty acids and alcohol, in sophorolipid fermentation culture allows for the production of sophorolipids with shorter fatty acid chain lengths as a consequence of surpassing the hydroxylation step in the sophorolipid biosynthetic process [21]. Furthermore, genetic engineering of native sophorolipid-producing strains via genetic knock-out of lactone esterase genes allows for the production of sophorolipids with different structural forms (e.g., bola-amphiphilic sophorolipids), degrees of acetylation, and/or acetyl chain lengths and, consequently, differential physiochemical and bioactivities [21,25,115]. Di-acetylated sophorolipids have been demonstrated to be less soluble and more cytotoxic than non-acetylated groups [59,120]. Therefore, in view of the fact that it is a priority of the cosmetic industry to investigate bioactive agents with little or no cytotoxic effects on the normal human skin and the skin microflora and with added functionalities, such as foaming and solubilisation, for incorporation into skincare products, non-acetylated sophorolipid would be desirable for use in skincare formulation [16,25,33,36,59,120].

**Figure 2:**
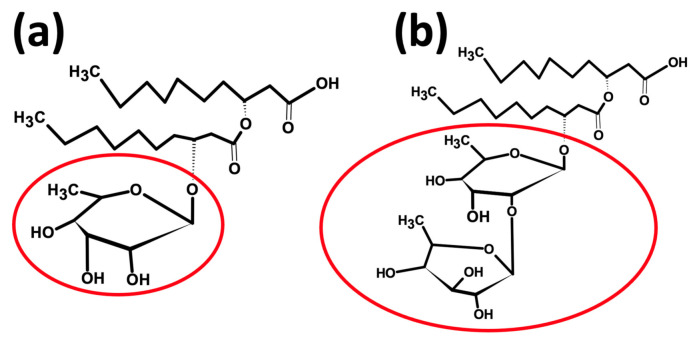
Chemical structure of (**a**) mono-rhamnolipids (one rhamnose as hydrophilic moiety) and (**b**) di-rhamnolipids (two rhamnose as hydrophilic moiety). Figure adapted from [19] and created with ChemSketch.com (accessed on 15 May 2023).

Unlike sophorolipids, up to 60 separate congeners of rhamnolipid molecules have been reported [2]. The diversity in the structure of the various rhamnolipid congeners is the result of the modifications in their hydrophilic and hydrophobic components, which are often occasioned by the diversity in rhamnolipid-producing strains, fermentation substrates, and culture conditions [2,121]. The commonest structural form of rhamnolipid molecule produced by bacteria is composed of one (mono-rhamnolipids) or two rhamnose (di-rhamnolipids) as hydrophilic moiety bonded to a hydrophobic moiety of one or two β-hydroxy fatty acid chains (8–16 carbon chain length) via an α-1,2-glycosidic linkage (Figure 2) [2,122,123]. However, depending on the number of 3-hydroxy fatty acid chains present, rhamnolipids can be further classified into four homologues, i.e., mono-rhamnolipids and di-rhamnolipids with two 3-hydroxy fatty acids are termed as mono-rhamno-**dilipids** and di-rhamno-**dilipids**, respectively, whereas mono-rhamnolipids and di-rhamnolipids with one 3-hydroxy fatty acid are termed mono-rhamno-**molipids** and di-rhamno-**molipids,** accordingly [23,124]. These groups of rhamnolipids are reported to be produced via the hydrolysis of one of the two 3-hydroxy fatty acid chains predominant in conventional mono-rhamnolipids and di-rhamnolipids by an unknown α/β-hydrolase enzyme [22,23].

Further alternatives to improving variation in rhamnolipid homologues could include a modification of their fatty acid chain lengths and branching, the number of l-rhamnose sugars, saturated/unsaturated bonds, and functional groups on the hydrophilic rhamnose heads of desired rhamnolipids for specific applications [2,23,124]. Notwithstanding this, it must be emphasised that as the properties of these glycolipids cannot be predicted from only their molecular structure, they should be evaluated experimentally for physiochemical and bioactivities. This will open the possibility to produce and investigate novel and diverse rhamnolipid homologues with known differential physiochemical and bioactivities for skincare applications.

### 3.2. Utilisation of Impure/Poorly Characterised Glycolipid Biosurfactant Congeners in Bioassays

Glycolipid biosurfactants are generally produced as a crude mixture of different congeners; however, these different congeners have been demonstrated to have varying bioactivities [33,36,120]. A significant number of studies on the bioactivities of glycolipids were performed using either a poorly characterised, a single class, or crude mixtures of different glycolipid congeners, whose purity and relative abundance of congeners present may not be reported [27,31,42,125,126,127]. Worthy of note is the assessment of antimicrobial effects of sophorolipids on a selection of Gram-negative and Gram-positive bacterial isolates using mixed preparations of sophorolipid congeners, whose methods of purification and chemical characterisation were not reported [126]. Although the mixed sophorolipid congeners were demonstrated to have significant inhibitory effects on the bacterial cells in both planktonic and biofilm states, the effects observed could not be attributed to any specific sophorolipid congeners [126]. More recently, Semkova et al. [121] investigated the anticancer and autophagy inhibitory effects of rhamnolipids, where the compounds were purified and separated into Mono-RL and Di-RL congeners. However, the only analytical technique utilised was thin layer chromatography (TLC) [127]. Despite the simplicity and reproducibility of the TLC analytical technique, it is non-quantitative and does not provide information about the congener profile of glycolipids [12,128]. Moreover, depending on the method of analysis and purity of the glycolipid extracts, there is a possibility of generating false-positive results [128]. Hence, the congener profile and purity of the rhamnolipids utilised by Semkova et al. in anticancer and autophagy assessments could not be determined, neither were the bioactivities observed conclusively discussed as a consequence of the potential congeners present [127].

The use of mixed, impure, and/or poorly characterised preparations of glycolipid congeners in bioassays often results in significant challenges, such as increased toxicity, contaminant interference with the bioactivities of glycolipids, inaccuracy in identifying the specific bioactivity of a particular glycolipid congener, and interstudy variations in biosurfactant research and applications [10,12,120]. In particular, in biosurfactant applications, an important drawback is the reduced desirability for the incorporation of glycolipids into skincare and pharmaceutical products given that in the formulation of cosmeceutical products, the careful selection of ingredients with known purity, chemical structure/composition, physiochemical properties, and bioactivities is a significant step towards ensuring the safety of the finished products [10,129]. Hence, Section 3 of the EU cosmetic legislation (2013/674/EU) requires that properties of cosmetic and skincare ingredients such as purity of compounds, molecular weight and structure, physiochemical properties, and concentrations utilised be assessed and reported by appropriate bodies/organisations [130]. It is clear, therefore, that to accurately determine the efficacy of individual glycolipid congeners and to broaden their potential applications in the skincare industry, purified and properly characterised preparations of glycolipid congeners should be utilised in bioassays [12,120].

At present, genetically modified glycolipid-producing strains and robust protocols for glycolipid purification and chemical characterisation are the focus for generating purified and chemically characterised glycolipid congeners [12,19,21,131,132]. For instance, sophorolipids produced by *S. bombicola* usually exist as a crude mixture of both lactonic and acidic congeners following lactonisation of intracellularly secreted Acidic SL congeners by the lactone esterase enzyme (sble) in the membrane of *S. bombicola* [133,134]. Therefore, the generation of −*Δsble S. bombicola* mutant strains offers the exclusive synthesis of Acidic SL in that the sophorolipids produced are not further lactonised while exiting the cell membrane [135]. Alternatively, alkaline hydrolysis of sophorolipid mixtures also results in the generation of a high proportion of Acidic SL [36,136]. Nevertheless, low product yield and prolonged solvent-based purification steps could make this alternative purification technique less desirable for use [136].

For rhamnolipids, efforts have been made to reduce the pathogenicity of the common producing strain, *P. aeruginosa*, via metabolic engineering and investigating a non-pathogenic alternative (*P. putida*) with enhanced yield capacity [41,104]. With regard to the generation of pure rhamnolipid congeners for academic research and industrial applications, a −*ΔrhlC P. aeruginosa* mutant strain has been generated to selectively produce Mono-RL due to the absence of the *rhlc* gene, which encodes the rhamnosyltransferase-2 (RhlC) enzyme to add a second dTDP-l-rhamnose molecule to the already-synthesised Mono-RL to form Di-RL [104,137,138]. Furthermore, organic solvents with differing levels of hydrophobicity and hydrophilicity could be utilised to separate mixtures of rhamnolipids into Mono-RL and Di-RL congeners via solid-phase extraction methods on a silica column (e.g., 55 μm, 70 Å strata SI-1 Silica giga tubes). The solid-phase extraction of rhamnolipid congeners is often preceded by the acidification of a rhamnolipid-rich cell culture supernatant to pH 2 to enhance rhamnolipid precipitation and subsequent liquid-phase extraction using ethyl acetate [110,139].

Once glycolipid biosurfactant congeners are extracted and purified, the next step is their chemical characterisation [128,139]. Current methods for characterising glycolipid biosurfactants range from the use of basic phenotypic testing (mainly to assess the surface activity of glycolipids) and colorimetric analyses, such as orcinol and anthrone reagent assays, to the use of highly sensitive chemical analytical techniques, such as nuclear magnetic resonance spectroscopy (NMR), gas chromatography (GC-MS), and HPLC/MS/ESI, all of which have been extensively reviewed by Twigg et al. [12]. Among these characterisation techniques, the chromatographic separation and characterisation technique, HPLC/MS/ESI, is rapid, cost-effective, and one of the most precise methods for analysing glycolipid congeners [12,35]. The HPLC/MS/ESI-glycolipid characterisation method utilises a combination of two robust chemical analytical techniques, i.e., HPLC separates glycolipids into their individual congeners using chromatographic columns, whereas the MS/ESI determines the masses of ions present in the congeners, which are compared with the literature for congener profile analysis and the identification of the molecular structure [35,68,128].

### 3.3. Limited In Vitro Studies on Potential Benefits of Glycolipids to the Human Skin and the Skin Microbiome and Treatment Conditions

To assess the potential to incorporate glycolipid biosurfactants into skincare products, their safety and bioactivities are generally investigated using in vitro assays with skin cells and skin bacteria [27,31]. Consequently, several studies have demonstrated the effects of glycolipid biosurfactants on various human skin cells and bacterial cell types [19,27,31,59,91]. Nonetheless, at present, only a few of these studies have investigated the potential benefits of glycolipid biosurfactants to the human skin and skin microbiome in vitro. These potential beneficial effects may include, but are not limited to, skin surface moisturisation, immunomodulation in diseased skin, wound healing, the selective inhibitory effects of glycolipids against skin cancers, and the maintenance or restoration of healthy skin microbiome [26,27,66,85,140,141].

For in vitro assays on the effects of glycolipids on skin bacteria, most studies focused on antimicrobial effects against skin pathogens rather than the effects of glycolipid congeners on healthy skin isolates [32,91,93]. Da Fontoura et al. [84] reported the maximum and minimum inhibitory concentrations of sophorolipid mixtures against *Streptococcus mutants*, *E. coli*, *Salmonella entérica*, *S. aureus*, and *Enterococcus faecium* at 500 and 2000 μg mL^−1^, respectively. In other studies similar to the antimicrobial effects of sophorolipids, uncharacterised crude rhamnolipids extracted from *P. aeruginosa* MR01 were demonstrated to have inhibitory effects against a wide range of Gram-positive bacterial isolates including *M. luteus* at 32 μg mL^−1^, *S. epidermidis* at 128 μg mL^−1^, *S. pneumonia* at 128 μg mL^−1^, *B. cereus* at 128 μg mL^−1^, and *B. subtilis* at 128 μg mL^−1^ [91]. However, no inhibitory effects were reported for Gram-negative *P. aeruginosa* MR01 and *E. coli* utilised in the same study [91]. Although the outcomes of the above studies are promising, these studies only focused on the antimicrobial effects of glycolipids and not their compatibility effects on healthy skin bacteria or the skin microbiome. An assessment of the compatibility effects of glycolipid biosurfactants on skin bacteria can be carried out in vitro via bacterial viability assays using selected representatives of the normal human microflora in individual or cocultured state. Moreover, in the foreseeable future, further studies on the effects of glycolipids on human skin chemistry, skin microbiome diversity, and metabolomics could be assessed in vivo [80].

With regard to studies on the effects of glycolipids on skin cells, the focus of most research has been to evaluate the effects of the glycolipids on cell viability and/or the pattern of cell death in various cell types, including primary and immortalised human keratinocytes and dermal fibroblasts [28,142]. Consequently, in our most recent publications, we aimed to investigate the added potential benefits of purified and fully characterised glycolipid biosurfactant congeners to the human skin as a step further to cytotoxicity assessments in comparison with SLES [19,33]. Spontaneously transformed immortalised human keratinocytes (HaCaT cells) and human malignant melanocytes (SK-MEL-28) were utilised as surrogates for healthy and diseased human skin, respectively [19,33]. In summary, it was demonstrated that highly purified and chemically characterised sophorolipid and rhamnolipid congeners had differential effects on the skin cells depending on the chemical structure. More specifically, Acidic SL and Mono-RL SLES had negligible cytotoxic effects in comparison with SLES in that while SLES significantly reduced the viability of HaCaT cells at concentrations above 60 µg mL^−1^, no cytotoxic effects were observed in Mono-RL- and Acidic SL-treated HaCaT cells at up to 300 and 500 μg mL^−1^, respectively [19]. Lactonic sophorolipid congeners (Lactonic SL) significantly reduced the viability of the SK-MEL-28 cells at concentrations not detrimental to the HaCaT cells (up to 40 µg mL^−1^), indicative of the potential of the purified Lactonic SL to target malignant melanoma for destruction should they be incorporated into sunscreen formulations [33]. Additionally, all the purified glycolipid congeners utilised demonstrated antimetastatic effects against the SK-MEL-28 cells [33]. Furthermore, Mono-RL and Di-RL modulated cytokine production in bacterial lipopolysaccharide-treated HaCaT cells, whereas Acidic SL and Lactonic SL significantly improved in vitro wound healing at treatment concentrations as low as 20 µg mL^−1^ [19].

It should be noted that none of the above-mentioned added functionalities were reported in cells treated with SLES. These findings suggest that the purified glycolipid congeners utilised here could offer a substitute to synthetic surfactants and, in addition, perform anticancer and immunopharmacological roles essential for the treatment of skin cancer and skin infections, such as psoriasis. Nevertheless, it is very likely that the differential bioactivities reported in the studies above would not have been this apparent if the glycolipids were utilised in their crude state or as a mixture of various congeners. Therefore, as previously discussed, to accurately substantiate the bioactivities of individual glycolipid congeners in bioassays, the use of well-separated, highly purified, and chemically characterised glycolipid congeners is highly recommended.

It is important to also indicate that although the overarching aim of most in vitro assays on glycolipid bioactivities and safety assessment for potential skincare applications is to investigate natural and biocompatible alternatives to synthetic surfactants, the majority of these studies lack sufficient experimental controls, such as the use of synthetic surfactants [27,42]. We, therefore, recommend the utilisation of synthetic surfactants as experimental controls in bioassays as this will allow for a comparative analysis of the cytotoxic effects between the synthetic surfactants and glycolipid biosurfactants.

Furthermore, considering that cytotoxicity is not predicated on the effects on cell viability and changes in cell morphology only, future studies should further investigate whether at non-inhibitory concentrations, the glycolipid biosurfactants will have non-deleterious effects on the production of inflammatory cytokines, necrotic and apoptotic cell-death induction, and the production of reactive oxygen species (ROS), using robust and reproducible experimental techniques. For instance, a combination of immunoassays, i.e., enzyme-linked immunosorbent assays (ELISA) and real-time quantitative PCR (qPCR), could be utilised for cytokine assays, whereas flow cytometry assays could be used to distinguish the pattern of cell death and the intracellular levels of ROS after glycolipid treatments [19,142].

Another important consideration for assessing the safety of glycolipid biosurfactant congeners for skincare applications using bioassays is the adherence to/utilisation of safety standards in cytotoxicity assessments [143]. Cytotoxicity is characterised by adverse effects on cells and tissues after exposure to a treatment agent(s) at known concentrations within a specified time [144,145]. Standard methods for assessing cytotoxic effects, therefore, require that the concentration of treatment agents be known as well as the accurate measurements of dose-dependent effects on cellular and tissue functions and the integrity within a specified time [144,145,146]. As such, researchers should endeavour to keep to the concentrations of synthetic surfactants mostly utilised in skincare products (0.01%–50% (*v*/*v*)) and the standards set out by the Organisation for Economic Co-operation and Development (OECD-Test no. 439), which recommends the use of up to 5% (*v*/*v*) sodium dodecyl sulphate (SDS) as positive control for cytotoxicity assays [56,146].

A well-known advantage of glycolipid biosurfactants over synthetic surfactants is their low critical micelle concentration (CMC) [26,147]. CMC is the concentration after which further increase in surfactant concentration will have no significant effects on the reduction of the surface and interfacial tension of fluids as surfactant monomers assemble themselves to form micelles [148,149]. Surfactants with low CMC are generally considered effective as less amounts would be required to form micelles and subsequently perform surface activities, such as foaming and emulsification, in skincare formulations [149,150,151]. The significant difference in CMC between glycolipids and synthetic surfactants has been reported in several studies, where the authors indicated that the CMC of sophorolipid and rhamnolipid congeners were at least tenfold lower than the CMC of the synthetic surfactants utilised in the same study; this demonstrates a potential preferential use of these glycolipids over synthetic surfactants in skincare applications as less amounts would be required to achieve the needed surface activity [19,152,153,154]. However, it should be acknowledged that glycolipids are readily biodegradable and can lose some activity with time depending on the concentration at which they are prepared, batch storage conditions, and variation in-house extraction/purification methods [155]. This poses significant questions for glycolipids in terms of shelf life, rheology (stability, spreadability/sprayability, etc.), and colour of skincare formulations should they be incorporated into skincare products, all of which are worth investigating further.

### 3.4. Utilisation of 2D In Vitro Cell Cultures in Bioassays

Using bioassays, glycolipid biosurfactants have been demonstrated to be a promising alternative to synthetic surfactants for use in skincare applications [19,59]. Notwithstanding, it is worth acknowledging that at present, most in vitro studies on the effects of glycolipid biosurfactants on the human skin and skin bacteria are performed using a monolayer of cells (2D in vitro cell cultures) [27,42,59,127]. These cells were utilised in their primary state or were spontaneously immortalised to escape cellular senescence and allow for indefinite proliferation while maintaining genetic and phenotypic identity close to the tissue of origin [156,157].

Although 2D in vitro cell culture models are cheap and easy to use and have contributed significantly to in vitro studies, they do not always provide an ideal representation of in vivo systems when utilised in drug safety and efficacy analyses considering that they are developed as a monolayer of cells on hard plastic surfaces, hence offering no opportunity for cell-to-cell or cell–matrix interactions prevalent in in vivo systems [158,159,160]. It is these cellular interactions that ensure controlled cell growth and enhanced physiological functions via molecular signalling in in vivo systems [159]. Thus, in vitro studies using 2D cell culture models may not translate into effective clinical trials as the response of a monolayer of cell culture to therapeutic agents differs with the in vivo systems [159,161]. Moreover, the use of animal models as a step further to the use of 2D cell cultures comes with significant limitations, such as ethical considerations and intrinsic differences in the anatomical structure, physiological functions, and host microbiota between human and animal models [162,163].

Therefore, in agreement with the EU directive (Council Directive 76/768/EEC) on a proposed ban on the use of animal models for cosmetic and pharmaceutical ingredient testing coupled with the limitations associated with the use of 2D in vitro cultures, the use of novel technologies to develop 3D in vitro skin models has become increasingly important [163,164,165]. The use of these 3D in vitro skin models allows for a pragmatic representation and appropriate mimicking of the complex anatomy and physiological functions of the in vivo and ex vivo human skin while providing an alternative to 2D in vitro skin cultures and the use of animal models in laboratory research [166].

**Figure 3:**
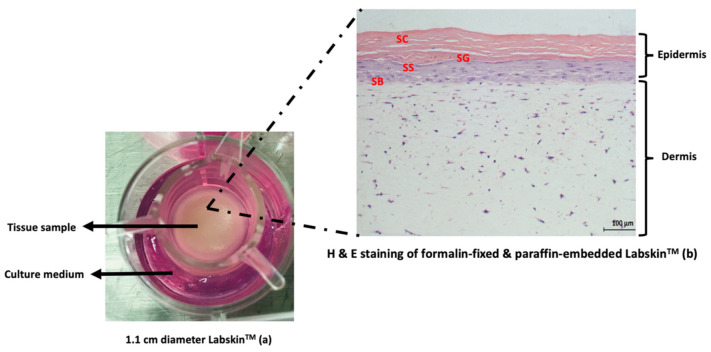
Experimental setup of (**a**) a 1.1 cm diameter Labskin^TM^ full thickness 3D in vitro skin model maintained on culture medium and (**b**) haematoxylin and eosin stain of Labskin^TM^ after formalin fixation, paraffin embedding, and microtome sectioning. Distinctive epidermal and dermal layers and the four main subepidermal layers, stratum corneum (SC), stratum granulosum (SG), stratum spinosum (SS), and stratum basale (SB), are identifiable.

At present, several 3D skin models are commercially available for testing the safety and efficacy of drugs and cosmeceutical ingredients [167,168]. Examples of commercially available 3D in vitro skin models include a Labskin^TM^ full thickness 3D in vitro skin model (Labskin^TM^), Episkin^TM^, MelanoDerm^TM^, and Phenion^TM^ FT LongLife skin model [168]. Established among these models is Labskin^TM^ [169,170]. Labskin^TM^ is a full thickness human skin equivalent specifically developed for studying interactions between the human skin and skin microbiome [171]. Compared with other 3D in vitro skin models, anatomically, Labskin^TM^ is composed of air-exposed epidermis comprising both primary human keratinocytes isolated from neonatal foreskin as the topmost layer and the dermal layer of polymerised fibrin containing adult human fibroblastic cells, thus providing the robust structural architecture of Labskin^TM^ (Figure 3) [170]. Moreover, the robust structural architecture of Labskin^TM^ in addition to its well-differentiated epidermis, barrier functions, dry acidic surface, and robust dermal layer makes it an ideal model for studying the effects of cosmetic ingredients on the interaction between the human skin and skin bacteria in vitro (Figure 3) [166,170].

Three-dimensional in vitro skin models have been utilised in a number of in vitro assays, including the assessment of skin barrier properties, examination of skin barrier repairs, processes of wound healing, immunoassays, and skin microbiome analyses following colonisation with a wide range of skin commensals [162,166,170,172,173]. However, to date, no study has investigated the effects of purified and chemically characterised glycolipid biosurfactant congeners on 3D in vitro skin models for potential skincare applications, to the best of the authors’ knowledge. Therefore, to appropriately substantiate and validate the efficacy and differential bioactivities of individual glycolipid congeners on the human skin and skin bacteria under conditions highly similar to the in vivo and ex vivo human skin and the skin microenvironment, we suggest the utilisation of a well-robust full thickness 3D in vitro human skin model in the safety assessment of purified glycolipid biosurfactant congeners. Consequently, this would broaden our understanding of the effects of glycolipids on the in vivo normal human skin and the skin microenvironment while progress is being made for potential future clinical trials.

## 4. Conclusions

Despite the promising potential benefits of sophorolipid and rhamnolipid biosurfactants and their subsequent use in several industrial applications, there are several challenges affecting the production of repeatable and comparable research outcomes in glycolipid-related academic research and for skincare applications, rendering these glycolipids economically unattractive and less competitive with synthetic surfactants. This review has discussed some of these challenges and reported relevant recommendations for future exploitation of sophorolipid and rhamnolipid biosurfactants. It is our hope that these measures, when strictly adhered to, could contribute significantly to increasing the acceptance of glycolipid biosurfactant for use in skincare applications while maintaining consistency in glycolipid-related research outputs.

## Figures and Tables

**Table 1:** A summary of both cellular and microbiome effects glycolipid-type biosurfactant molecules elicit on or within the human skin.

Glycolipid Subclass	Effect	References
All glycolipids	Affect surface chemistry of the skin, promoting skin commensal bacteria	[85,86,87,88]
	Provide antibiotic synergy against Gram-negative pathogens	[54,94,95]
Rhamnolipids	Antimicrobial effects on Gram-positive bacteria	[93]
	No detrimental effects on human HaCaT keratinocyte cell line	[33,38]
	Detrimentally affect SK-Mel-28 melanoma cell line	[33]
Sophorolipids	Antimicrobial effect on *Staphylococcus aureus*	[92]
	Antimicrobial effect on *Streptococcus pyogenes*	[92]
	Antimicrobial effect on *Cutibacterium acnes*	[92]
	Accelerate dermal wound healing in vitro	[27]
	Stimulate *Col-1* gene expression	[27]
	Inhibit elastase enzymes	[27]
	No detrimental effects on different healthy human skin cell lines	[27,33,59]
	Attenuate gene expression of proinflammatory cytokines	[27]
	Detrimentally affect SK-Mel-28 melanoma cell line	[33]
Uncharacterized glycolipids	Antimicrobial effect on *Staphylococcus aureus*	[97]
	Antimicrobial effect on *Staphylococcus epidermidis*	[97]
	Antimicrobial effect on *Pseudomonas aeruginosa*	[97]
	Antimicrobial effect on *Salmonella typhi*	[97]
	Antimicrobial effect on *Escherichia coli*	[100]
	Antimicrobial effect on *Bacillus subtilis*	[100]
